# Tiron Inhibits UVB-Induced AP-1 Binding Sites Transcriptional Activation on MMP-1 and MMP-3 Promoters by MAPK Signaling Pathway in Human Dermal Fibroblasts

**DOI:** 10.1371/journal.pone.0159998

**Published:** 2016-08-03

**Authors:** Jing Lu, Jia-Hui Guo, Xue-Liang Tu, Chao Zhang, Mei Zhao, Quan-Wu Zhang, Feng-Hou Gao

**Affiliations:** 1 Institute of Oncology, Shanghai 9th People's Hospital, Shanghai Jiao Tong University School of Medicine, Shanghai, China; 2 Yellow River Hospital Attached Henan University of Science and Technology, Sanmeixia, China; 3 Department of Reproductive Medicine, Shanghai First Maternity and Infant Hospital, Tongji University School of Medicine, Shanghai, China; 4 Department of pathology, Zhengzhou central hospital affiliated to Zhengzhou University, Zhengzhou, China; San Gallicano Dermatologic Institute, ITALY

## Abstract

Recent research found that Tiron was an effective antioxidant that could act as the intracellular reactive oxygen species (ROS) scavenger or alleviate the acute toxic metal overload in vivo. In this study, we investigated the inhibitory effect of Tiron on matrix metalloproteinase (MMP)-1 and MMP-3 expression in human dermal fibroblast cells. Western blot and ELISA analysis revealed that Tiron inhibited ultraviolet B (UVB)-induced protein expression of MMP-1 and MMP-3. Real-time quantitative PCR confirmed that Tiron could inhibit UVB-induced mRNA expression of MMP-1 and MMP-3. Furthermore, Tiron significantly blocked UVB-induced activation of the MAPK signaling pathway and activator protein (AP)-1 in the downstream of this transduction pathway in fibroblasts. Through the AP-1 binding site mutation, it was found that Tiron could inhibit AP-1-induced upregulation of MMP-1 and MMP-3 expression through blocking AP-1 binding to the AP-1 binding sites in the MMP-1 and MMP-3 promoter region. In conclusion, Tiron may be a novel antioxidant for preventing and treating skin photoaging UV-induced.

## Introduction

Fibroblasts are the most common connective tissue cells, coming from differentiation of embryonic mesenchymal cells [1]. Fibroblasts have a very important reparative effect on the cell degeneration, necrosis, trauma and tissue defect of different degrees. Studies have shown that fibroblasts in the lower layer of skin connective tissue are responsible for regulating extracellular matrices, interstitial fluid volume and pressure, and wound healing [2, 3]. Also found that change in collagenous ECM is a most prominent feature of skin connective tissue aging, manifested as skin collagen, hyaluronic acid and elastin degradation, loss of skin elasticity, which makes skin become rough, as well as easy to form wrinkles [4, 5]. More importantly, these changes can cause the elderly skin prone to injury, but hard to heal.

Cellular change as well as qualitative and quantitative alterations of dermal extracellular matrix proteins is the main cause of the elderly skin senescence [6]. On the one hand, lack of number of skin fibroblasts could not meet the requirements of the normal and effective repair; on the other hand, the existing aging skin fibroblasts not only fail to work, but even have the adverse effect. For example, it has been found that these aging fibroblasts could synthesize and secrete large amounts of matrix metalloproteinases 1 and 3(MMP-1 and MMP-3) to degrade skin collagen matrix to accelerate the progression of skin aging [7]. Faced with these unfavorable factors, we should look for some effective approaches to stimulate the growth of skin fibroblasts; at the same time, we must seek methods to suppress the aging fibroblasts synthesizing and secreting matrix metalloproteinases (MMPs). MMPs are a family of structurally related matrix degrading enzymes which play critical roles in various destructive processes including tumor invasion, inflammation and skin aging [8, 9]. Only if these two aspects have been taken effective measures simultaneously, it is very likely to change the elderly skin and restore skin elasticity, as well as to speed up the wound healing. Because the direct cause of skin fibroblasts aging is subjected to its long-term oxidative stress in vivo, previous researches about improving the quantity and quality of skin fibroblasts were mainly focused on the application of some chelating agents to make the local skin oxidative stress microenvironment better, thus contributing to increase in proliferation and functional recovery of skin fibroblasts [10].

Tiron, 4, 5-dihydroxy-1, 3-benzene disulfonic acid, with the water-soluble and cell permeable characteristic, is an effective antioxidant, that can remove all kinds of harmful free radicals [11]. For example, it can reverse the reactive oxygen species (ROS)-induced cell apoptosis, or act as a nontoxic chelating agent to alleviate the acute toxic metal overload [12]. Tiron has been reported to be able to inhibit apoptosis in human lung cancer cells induced by bortezomib [13]. In addition, it was reported that it can significantly inhibit melatonin-induced superoxide dismutase (SOD) activation in mitochondrial intermembrane space in rats [14]. Tiron and selenium combinative usage is an effective treatment to prevent and reduce the extent of vanadium poisoning in rats [15]. Previously reported that Tiron could rapidly and strongly bond with superoxide anion, followed by reducing the toxicity of oxidative stress, thereby inhibiting UVB-induced human skin fibroblasts senescence [16, 17].

Here, Tiron is applied to the ultraviolet-induced skin fibroblasts photoaging model to observe its effect on matrix metalloproteinases synthesis and make further efforts to explore the underlying molecular mechanisms of Tiron delaying photoaging in fibroblasts, which provides the experimental basis for the application of Tiron to restore the function of skin fibroblasts.

## Materials and Methods

### Ethics Statement

This study was approved by the Research Ethics Committee of Shanghai 9th People's Hospital, Shanghai Jiao Tong University School of Medicine. We obtained their informed consent from the next of kin, caretakers, or guardians on behalf of the minors/children in our study. The consent was verbal because the skin tissue we used was from the discarded resource in the surgery process. Thus, the requirement for written informed consent was waived.

### Reagents

Tiron was obtained from Sigma (St. Louis, MO). High glucose-containing DMEM, FBS and PBS were obtained from Gibco-BRL (Gaithersburg, ME).

### Isolation and culture of HDF

Human dermal fibroblasts were aseptically isolated from a circumcised neonatal foreskin. The epidermis and dermis were separated by incubation in 0.9 units/ml dispase in medium for 16 h at 4°C. After the epidermis and dermis were mechanically separated, the dermis was minced and attached on the surface of tissue culture flask and fed with DMEM containing 10% FBS for 1–2 weeks. The dermal fibroblasts spreading as radial outgrowth from attached pieces of dermis, were cultured in DMEM with 10% FBS.

### UV irradiation

For a UVB irradiation, we used UVB cross-linker (6 × 8 W, 312 nm, Model CL-508M, Vilber lourmat, Paris, France). In brief, serum-starved confluent cells were rinsed twice with PBS, and all irradiations were performed under a thin layer of PBS. Immediately after irradiation, fresh serum-free medium was added to the cells. Responses were measured after an incubation period of 24, 48, 72h, respectively. Mock-irradiated controls followed the same schedule of medium changes without UVB irradiation.

### Real Time polymerase chain reaction

Total cellular RNAs were extracted from cells by TRIzol reagent (Invitrogen, CA). Real Time polymerase chain reaction was performed with PrimeScript RT reagent Kit with gDNA Eraser (Takara, JA). Reverse transcription was performed in 10 μl volume, starting with 500 ng of total RNA. The reaction mixture was initially heated to 37°C for 30 min, 85°C for 5 sec and finally to 4°C for 5 min. In the PCR step, PCR products were amplified from cDNA samples using specific primers and all assays were performed in triplicate on the LightCycler480 (Roch, CH). The primers used were: MMP-1, 5'-CAT CGT GTT GCA GCT CAT GA-3'(forward), and 5'-ATG GGC TGG ACA GGA TTT TG -3'(reverse); MMP-3,5'-TGC TGC TCA TGA AAT TGG CC-3'(forward), and 5'-TCA TCT TGA GAC AGG CGG AA -3' (reverse); actin, 5'- TCG TGC GTG ACA TTA AGG AG -3' (forward), and 5'- GTC AGG CAG CTC GTA GCT CT -3' (reverse). The assay tubes were initially heated to 95°C for 30 sec, followed by 40 cycles of 95°C for 5 sec and 60°C for 60 sec. The dissociation curve of each amplification production was analyzed to confirm that there were no non-specific PCR products. The expression levels of candidate RNAs were normalized using actin as the endogenous control. Relative quantitative expression levels of RNAs were determined by the 2-△△CT method.

### Determination of MMP-1 and MMP-3 secretions by ELISA

Secretion activity of MMP-1 and MMP-3 were detected by ELISA kit under the manufacturer’s instruction (Cusabio, USA). Remove particulates by centrifugation for 15 minutes at 1000xg, 2–8°C and assay immediately or aliquot and store samples at -20°C or -80°C. Add 100 μl of standard and sample per well and incubate for 2 hours at 37°C. Remove the liquid of each well, don’t wash. Add 100 μl of Biotin-antibody (1x) to each well and incubate for 1 hour at 37°C. Wash by filling each well with Wash Buffer for 3 times. Add 100 μl of HRP-avidin (1x) to each well and incubate for 1 hour at 37°C. Add 90 μl of TMB substrate to each well and incubate for 15–30 minutes at 37°C. Add 50 μl of stop solution to each well, gently tap the plate to ensure thorough mixing. Determine the optical density of each well within 5 minutes, using a microplate reader set to 450 nm.

### Western blotting

Cells were washed once with PBS and then lysed in buffer containing 100 mM Tris-HCL (pH 6.8), 200 mmol/L DTT, 4% SDS, 20% glycerine. Protein concentrations in the supernatants were determined by the Bradford method. Equal amounts of protein from whole-cell lysates were separated by gel electrophoresis on 10% gels, transferred to PVDF membranes and were probed with specific primary antibody [MMP-1goat mAb; MMP-3 mouse mAb; Pi-ATF-2 mouse mAb, ATF-2 rabbit mAb (Santa Cruz, CA), Pi-Erk rabbit mAb, Erk rabbit mAb, Pi-Jnk rabbit mAb, Jnk rabbit mAb, Pi-P38 rabbit mAb, p38 rabbit mAb, Pi-c-Jun rabbit mAb, c-Jun rabbit mAb, C-Fos rabbit mAb, Pi-C-Fos rabbit mAb (Cell Signaling, MA) and then with the appropriate HRP-conjugated secondary antibodies. Proteins were detected using the enhanced chemiluminescence detection kit (Thermal Science, USA). For loading control, the membrane was probed with a monoclonal antibody for GAPDH (Kangchen Biotechnology, Shanghai). The quantitative analysis of the proteins was performed by normalizing against GAPDH or total protein by using Image Pro Plus 6.0 software (Media Cybernetics, Inc., Silver Spring, MD, USA).

### Plasmid construction and transfection

Promoter and 5’UTR of MMP-1/MMP-3 was amplified using genomic DNA with primers. The primers used were: MMP-1,5'-gcc ctc gag tct ggg att ata ggc ttg a-3'(forward), 5'-cgc aag ctt act ggc ctt tgt ctt ctt-3'(reverse); MMP-3,5'-gcc ctc gag cca gca aat cca acg aca-3'(forward), 5'-cgc aag ctt ttc cac tgg ctt tac tta g-3'(reverse). The target fragments were purified, subcloned into pGL4.27 vector (BioVector, China) and sequenced. Sequence homologies were analyzed using NCBI’s BLAST. 293T cells were transfected with pGL4.27-MMP-1/MMP-3 promoter plasmids for 48 hours with Lipofectamine 3000 reagent (Invitrogen, USA) according to the manufacturer's instructions before UVB irradiation or Tiron treatment.

### Site-directed mutagenesis of binding sites in MMP-1/MMP-3 promoter

The binding sites in the MMP-1/MMP-3 promoter constructs were mutated using Hieff MutTM Site-Directed Mutagenesis Kit (Yeasen, USA). The primers used were: MMP-1promoter-mutation, 5'-ATA AAA TGC AGA CTG GAC AGC CTC TGG CTT TCT GGA AGG-3'(forward), 5'-GCT GTC CAG TCT GCA TTT TAT AAC ATC CTC TTG ATT AGC TAT G-3'(reverse); MMP-3promoter-mutation, 5'-AGC AAA TGC AGA CTG AGC TGC GGG TGA TCC AAA CAA AC-3'(forward), 5'-CAG CTC AGT CTG CAT TTG CTT TCA TCC AAA TGG CAG CAG-3'(reverse).The mutations were confirmed by DNA sequencing.

### Luciferase reporter assay

The cells were plated in 6-well plates (3–5×104 cells per well) in triplicate for each condition. After overnight incubation, cells were transfected with the different DNA mix. Cells were treated with UVB irradiation and/or Tiron 48h post-transfection. Luciferase activities were measured 48h post-treatment using a Dual-luciferase reporter kit (Beyotime Biotechnology, China). Each experiment was performed in triplicate and repeated three times.

### Statistical Analysis

All experiments were repeated a minimum of three times. Data was analyzed by the difference between means, and statistical significance was calculated using Fisher’s least significant difference (LSD) or Student’s t-test. P<0.05 was considered to indicate a statistically significant difference.

## Results

### Tiron could reverse UVB-induced upregulation of MMP-1 and MMP-3 in HDFs

**Fig 1:**
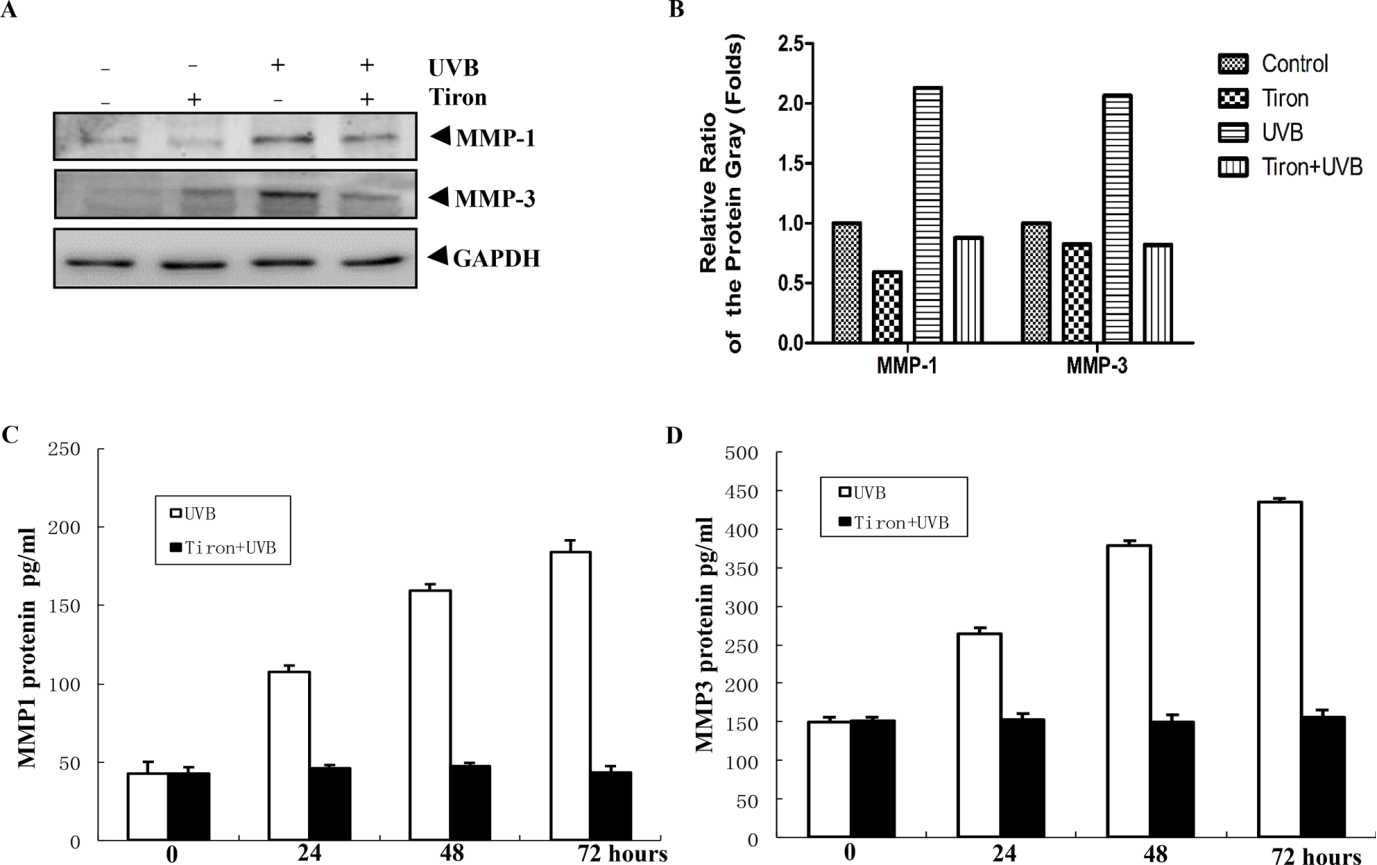
Tiron reverses the upregulation of MMP-1 and MMP-3 protein in UVB-irradiated HDFs. **A,** HDFs were treated with UVB40 mJ/cm^2^ alone or together with 0.2 mM Tiron 2h before the UVB exposure. HDFs were harvested 48h after these treatments. The expression of MMP-1 and MMP-3 was assayed by western blotting. **B**, The densitometric analysis for the data shown in (A) for MMP-1 and MMP-3 is shown. **C-D**,Then the pro-MMP-1 and MMP-3 secretions in cell supernatants were assayed at 0, 24, 48, 72h after the UVB treatment by ELISA. The error bar represents means ± S.E.M. (compared with the control group, *p* < 0.05).

Ultraviolet irradiation can damage human skin and promote collagen degradation, which eventually lead to premature skin aging (photoaging) [18, 19]. Some studies have proved that ultraviolet radiation can induce the expression of MMPs, which are able to degrade the collagen, one of major components in the extracellular matrix [20, 21]. Among them, MMP-1 and MMP-3 are considered as two major contributors to photoaging [20]. Here, we used UVB 40 mJ/cm^2^ to irradiate HDFs, and then detected at 0, 24, 48, 72h post-irradiation respectively. It was found that expression of MMP-1 and MMP-3 increased gradually in a time-dependent way, and reached a peak at 72 h phase (S1A and S1B Fig). When we treated cells with Tiron alone, we found that Tiron made little difference to expression of MMP-1 and MMP-3 in HDFs (Fig 1A and 1B). However, if cells were treated with Tiron before UVB exposure, expression of MMP-1 and MMP-3 decreased greatly compared with the single UVB exposure group (Fig 1A and 1B). At the same time, we detect MMP-1 and MMP-3 secretions in supernatants through ELISA assays. Consistent with the previous results, expression of MMP-1 and MMP-3 both showed a rising trend at 24, 48, 72h post-irradiation (Fig 1C and 1D). This phenomenon showed the most obviously at 72h after irradiation. But if cells were got UVB radiation together with the Tiron pretreatment, MMP-1 and MMP-3 in the supernatants had almost no change in every period of time (Fig 1C and 1D). These experimental results show that while UVB radiation can upregulate remarkably the expression of MMP-1 and MMP-3 in HDFs in vitro, Tiron is able to reverse this influence, suggesting that Tiron may have the potential of anti-light aging effect.

### Tiron can inhibit the increasing mRNA levels of UVB-induced MMP-1 and MMP-3

**Fig 2:**
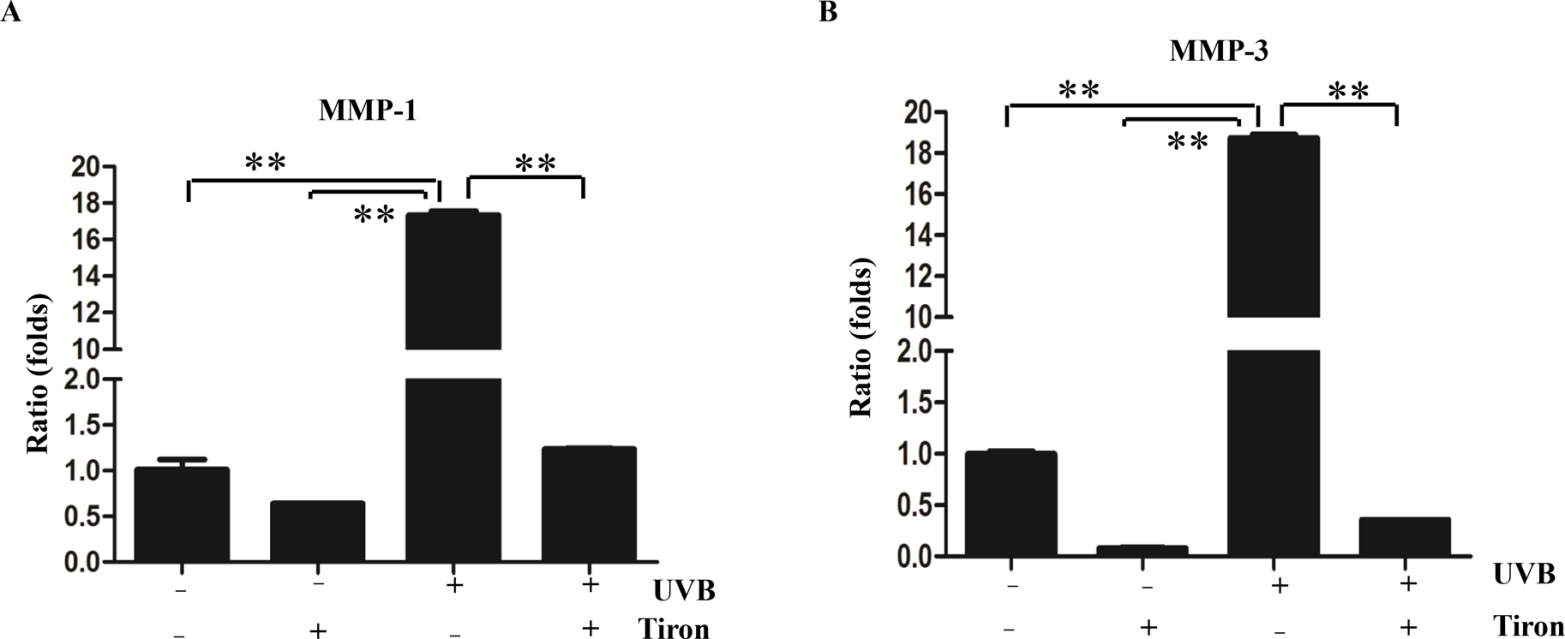
Effect of Tiron on UVB-induced MMP-1 and MMP-3 mRNA expression in HDFs. **A-B,** HDFs was treated with 0.2 mM Tiron, UVB40 mJ/cm^2^ alone or treated with 0.2 mM Tiron 2h before the UVB exposure. HDFs were harvested 48h after these treatments. RT-PCR was performed to detect MMP-1 and MMP-3 mRNA expressions. (**, *p* < 0.01).

Since protein levels of MMP-1 and MMP-3 can be upregulated dramatically by UVB and suppressed by treatment with Tiron, the effect of Tiron on gene expression should be further investigated. We want to know whether UVB increases the expression of MMP-1 and MMP-3 at the transcriptional level or at the post-transcriptional level from the perspective of molecular mechanisms. Therefore, mRNA levels of MMP-1 and MMP-3 were also detected after UVB irradiation with or without the Tiron pretreatment. As a result, a higher mRNA level of MMP-1 and MMP-3 was observed after the UVB irradiation. Furthermore, MMP-1 and MMP-3 mRNA was induced in a time-dependent manner, and the most significant induction was at 72h post-irradiation (S1C and S1D Fig). In another separate experiment, we observed that there was little change to mRNA expression if cells were only treated with Tiron (Fig 2A and 2B). However, while mRNA levels of MMP-1 and MMP-3 greatly increased after the UVB irradiation, they could be blocked by the Tiron (Fig 2A and 2B).

### Tiron can inhibit UVB-induced phosphorylation of MAPK signaling pathway

**Fig 3:**
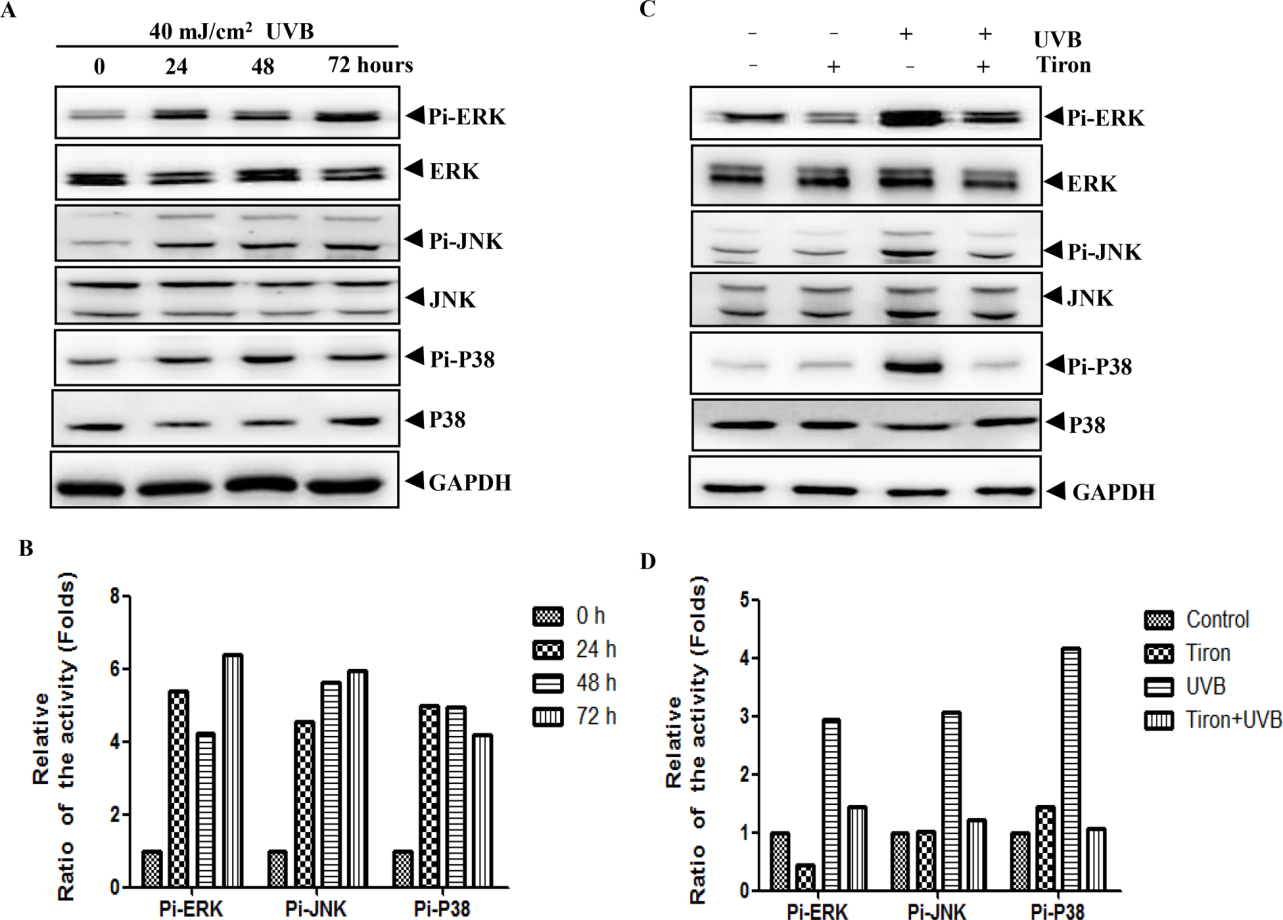
The UVB-induced phosphorylation level of the MAPK signaling pathway is inhibited by Tiron. **A,** HDFs were exposed to UVB light with a total dose of 40 mJ/cm^2^.The phosphorylation and total protein expression of the MAPK signaling pathway were assayed after the UVB treatment (0, 24, 48, 72h) by western blotting. Protein expression levels were normalized to that of GAPDH. **B**, The densitometric analysis for the data shown in (A) for Pi-ERK, Pi-JNK and Pi-P38 is shown. **C**, HDFs was treated with 0.2 mM Tiron, UVB40 mJ/cm^2^ alone or treated with 0.2 mM Tiron 2h before the UVB exposure. HDFs were harvested 48h after these treatments. The phosphorylation and total protein expression of the MAPK signaling pathway were assayed by western blotting. Protein expression levels were normalized to GAPDH. **D**, The densitometric analysis for the data shown in (C) for Pi-ERK, Pi-JNK and Pi-P38 is shown.

The MAPK signaling pathway is an important internal transmitter, responsible for transferring extracellular stimuli into the cell nucleus [22], which plays a key role in the regulation of gene expression, cell proliferation, differentiation, apoptosis and other life activities [23, 24]. The MAPK signaling pathway contains three important subfamilies, ERK, JNK and P38, respectively [25]. Previous studies have demonstrated that MAPK signaling pathway is one of the important activated signal transduction pathways to regulate cellular activities when exposed to the UVB irradiation [26, 27]. When skin is exposed to UVB, it will produce large amounts of ROS in cells, which can activate the MAPK signaling pathway, followed by downstream transcription factors phosphorylated to promote expression of MMPs, and finally leading to skin photoaging [28, 29]. Since Tiron takes an effect on reducing expression of MMP-1 and MMP-3, so we speculate that Tiron may work out through inhibiting the MAPK signaling pathway activation. To confirm this hypothesis, we first used UVB irradiate HDFs and then detected the total protein and phosphorylation levels of the MAPK signaling pathway at 0, 24, 48, 72h post-irradiation. We observed that there is little change to total protein levels of ERK, JNK and P38, but phosphorylation levels of ERK, JNK and P38 were obviously increased from 24h to 72h post-irradiation compared with the UVB untreated group (Fig 3A and 3B). However, if cells were treated with Tiron 2h before the UVB irradiation, the phosphorylation level of the MAPK signaling pathway would show a decreased state (Fig 3C and 3D). Wherein, phosphorylation inhibitory effect of p38 was the most obvious, and phosphorylation of ERK and JNK was just partially inhibited (Fig 3C and 3D). If cells were treated with Tiron alone, the phosphorylation level of the MAPK signaling pathway was similar to that of the untreated group (Fig 3C and 3D).

### Tiron can inhibit AP-1 activation in UVB-irradiated HDFs

**Fig 4:**
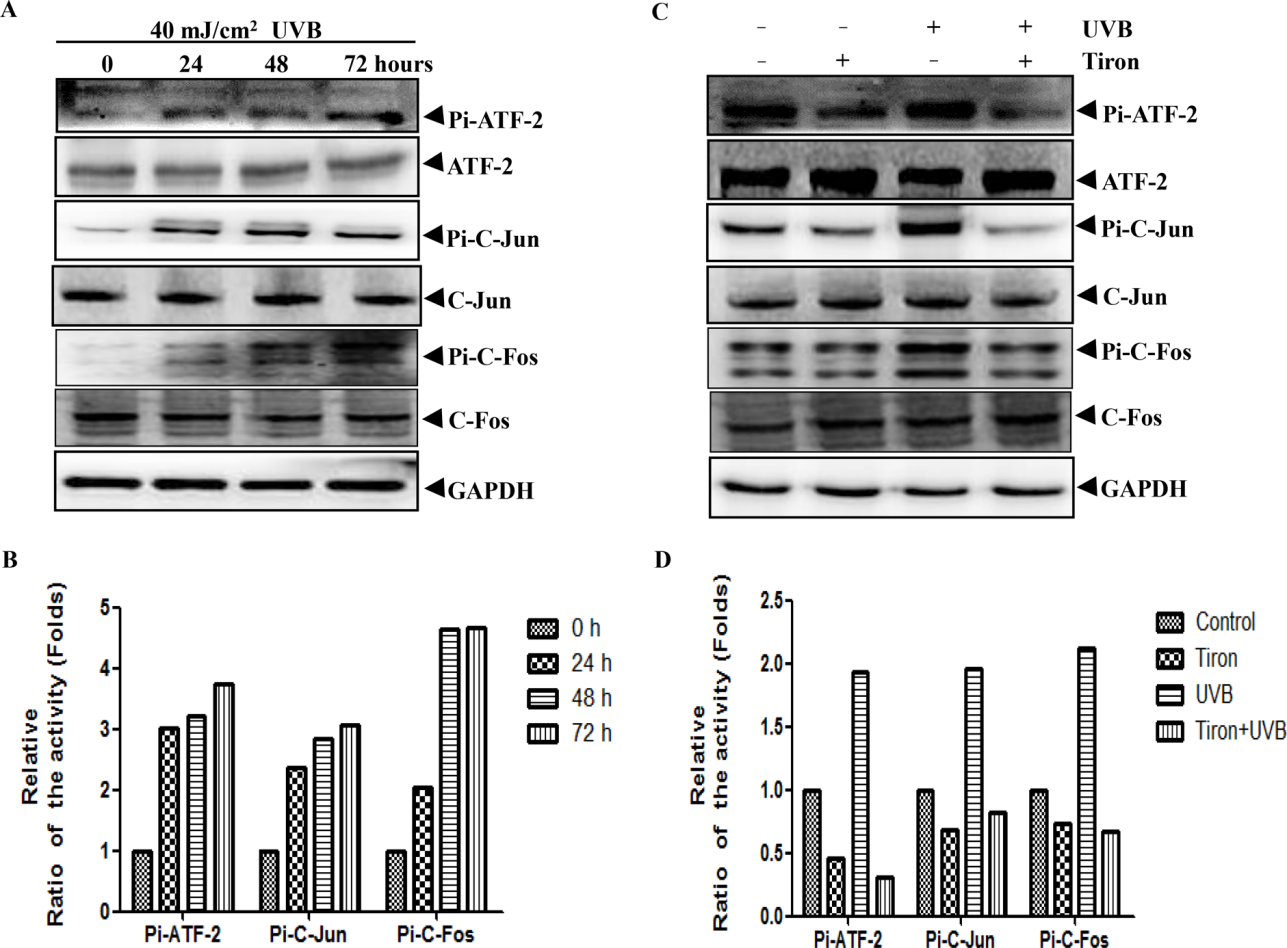
The UVB-induced phosphorylation levels of ATF-2, C-Jun and C-Fos from HDFs is inhibited by Tiron. **A,** HDFs were exposed to UVB light with a total dose of 40 mJ/cm^2^.The phosphorylation and total protein expression of transcription factors ATF-2,C-Jun and C-Fos were assayed after the UVB treatment (0, 24, 48, 72h) by western blotting. Protein expression levels were normalized to that of GAPDH. **B**, The densitometric analysis for the data shown in (A) for Pi-ATF-2, Pi-C-Jun and Pi-C-Fos is shown. **C,** HDFs was treated with 0.2 mM Tiron, UVB 40 mJ/cm^2^ alone or treated with 0.2 mM Tiron 2h before the UVB exposure. HDFs were harvested 48h after these treatments. The phosphorylation and total protein expression of ATF-2, C-Jun and C-Fos were assayed by western blotting. Protein expression levels were normalized to that of GAPDH. **D**, The densitometric analysis for the data shown in (C) for Pi-ATF-2, Pi-C-Jun and Pi-C-Fos is shown.

AP-1 is a downstream effector of the MAPK signaling pathway [30], which can cause degradation of collagen fibers by increasing MMPs expression [31–33]. Predictably, UVB-mediated skin photoaging can be prevented by the suppression of AP-1 activation [34]. To further investigate this mechanism of the transcription factors involved in regulating MMPs expression in HDFs, we examined the effect of Tiron on the activity of the AP-1 complex protein. AP-1 is a nuclear transcription complex composed of c-Jun and c-Fos [35, 36]. Moreover, in response to UV radiation, ATF-2 will be phosphorylated and activated simultaneously [37, 38]. Activated ATF-2 and C-Jun can compose of heterodimers to bind 'AP-1-like' sites initiate the transcription process and regulate gene expression [39]. Not surprisingly, although total protein levels of c-Jun, c-Fos and ATF-2 had little change, phosphorylation levels were gradually increased in a time-dependent manner between post-irradiation 0h and 72h (Fig 4A and 4B). If HDFs were treated with Tiron alone, both total protein levels and phosphorylation levels of signaling molecules did not changed obviously (Fig 4C and 4D). However, compared with the single UVB-treated group, phosphorylation levels of c-Jun, c-Fos and ATF-2 were significantly inhibited in the UVB and Tiron-treated group (Fig 4C and 4D). At the same time, total protein levels of all signal molecules did not changed greatly either (Fig 4C and 4D).These data suggested that Tiron played an inhibitory effect on AP-1 activation.

### AP-1 binding sites in MMP-1 and MMP-3 promoter region involve in Tiron to restrain their transcriptional activity UVB-induced

**Fig 5:**
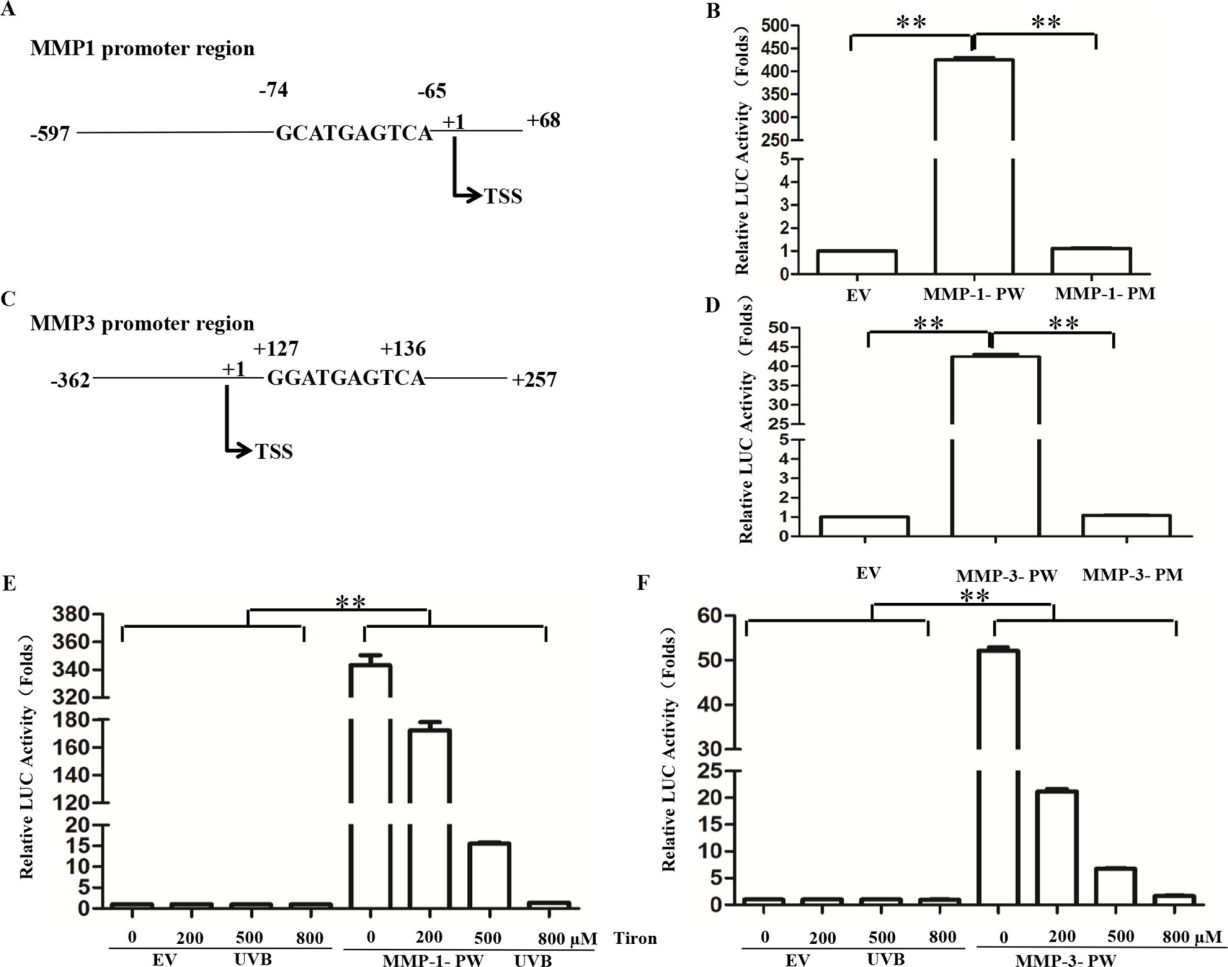
Tiron inhibits MMP-1 and MMP-3 expression by inhibiting AP-1 directly binding to promoter of MMP-1/MMP-3. **A-C,** According to the binding sites predicted by BIOBASE Biological Databases, the direct binding sites is likely located at TSS (**-**74 GCATGAGTCA**-**65) of the MMP1 promoter region and located at TSS (**+**127 GGATGAGTCA**+**136) of the MMP3 promoter region; TSS, transcription start site. **B, D-F,** The GCATGAGTCA sequence of the MMP1 promoter region was mutated into TGCAGACTGG; the GGATGAGTCA sequence of the MMP3 promoter region was mutated into ATGCAGACTG. The partial native AP-1 transcription factor-binding sites in MMP-1/MMP-3 promoter region and sites-mutated regions of the MMP-1/MMP-3 promoter were constructed into luciferase reporter EV, empty vector; MMP-1- PW, wild-type MMP-1 promoter; MMP-1-PM, mutated MMP-1 promoter; MMP-3- PW, wild-type MMP-3 promoter; MMP-3-PM, mutated MMP-3 promoter. The transfected 293T cells were treated with UVB or Tiron of different dose. The luciferase reporter assay was performed. (**, *p* < 0.01).

Futhermore, we searched the putative AP-1 binding sites in MMP-1/MMP-3 promoter region using BIOBASE Biological Databases (http://www.biobase-international.com/gene-regulation). Several AP-1-binding sites were predicted in MMP-1/MMP-3 promoter region. Regarding the distance between regulated genes and binding sites, we speculated that the nearest predicted sites may be the binding sites of target genes. MMP-1/MMP-3 promoter region were purified and subcloned into pGL4.27 vector. Then these sites in MMP-1 (-74GCATGAGTCA-65)/MMP-3 (+127GGATGAGTCA+136) promoter region were mutated (MMP-1, TGCAGACTGG; MMP-3, TGCAGACTG) and lost the AP-1 binding ability, after that mutated promoter regions were purified and subcloned into pGL4.27 vector (Fig 5A and 5C). To examine our hypothesis, we performed the luciferase reporter assays. Then plasmids were transfected into 293T cells, followed by treatment of UVB irradiation and/or Tiron. The data showed that after the mutated plasmid or empty vector was transfected, luciferase activity was not changed after UVB irradiation, while transfected with wild type promoter region plasmids, the luciferase activity was dramatically enhanced (Fig 5B and 5D). Interestingly, luciferase activity gradually decreased in a dose dependent manner in cells treated with Tiron (Fig 5E and 5F). These findings demonstrated that Tiron can inhibit MMP-1/MMP-3 expression via AP-1 binding sites on MMP-1/MM-P-3 promter region.

## Discussion

Some research have shown that the aging fibroblasts not only produce less extracellular matrix, but also able to secrete MMP-1 and MMP-3 to degrade matrix [20]. Upregulated MMP-1 and MMP-3 could accelerate the process of degradation of collage, leading to the result that integral collagen fibers gradually decreased and content of mature collagen in the skin is also reduced [40]. Here we find that Tiron could inhibit activation of the MAPK signaling pathway, thereby which inhibits transcription factor AP-1 mediated transcription of MMP-1 and MMP-3 in the context of UVB-induced fibroblast cell senescence model.

On one hand, the treatment of low-dose UVB (40 mJ/cm^2^) can lead to MMP-1 and MMP-3 protein expression gradually increased at 24, 48, 72h after irradiation, respectively, both in the cell lysates and supernatants of skin fibroblasts. On the other hand, UVB-induced upregulation of MMP-1 and MMP-3 could be obviously blocked in the fibroblasts when the cells were pretreated with Tiron. These results indicated that Tiron was able to interfere with the expression of MMP-1 and MMP-3 to regulate the rebalancing process of fibroblasts matrix. After that, we detected mRNA levels of MMP-1 and MMP-3 after treatment with UVB with or without Tiron pretreatment by real time RT-PCR, the data showed that mRNA levels of MMP-1 and MMP-3 were significantly increased after UVB treatment, while the Tiron pretreatment can inhibited UVB-induced upregulation. This suggested that UVB could increase protein levels of MMP-1 and MMP-3 by the way of transcriptional activation; meanwhile, Tiron was able to reverse UVB-mediated increase of MMP-1 and MMP-3 in the transcriptional level.

Emerging research showed that the MAPK signaling pathways are activated in fibroblasts when irradiated with UVB [41]. Thus, we speculated that whether Tiron could inhibit UVB-induced MAPK signaling pathway activation in fibroblasts, following impairing downstream transcription factors activation of signaling pathways, which finally contribute to UVB-induced expression of MMP-1 and MMP-3 showing a low-level expression state. Western blotting confirmed that after UVB irradiation, total protein levels of ERK, JNK and p38 were unchanged while their phosphorylation levels gradually increased in a time-dependent manner; whereas if pretreated with Tiron 2 hours before irradiation, phosphorylation levels of ERK, JNK and p38 were significantly downregulated compared with the single UVB irradiation treatment group. Considering the MAPK signaling pathway usually regulate gene expression through activation of associated transcription factors [42], so we detected total protein levels and their phosphorylated protein levels of c-Jun, c-Fos and ATF-2 after irradiated with UVB with or without the Tiron pretreatment in fibroblasts. As we expected, Tiron could obviously inhibit UVB-induced upregulation of their phosphorylation levels under the circumstance of not influencing the total protein levels. Collectively, UVB is able to activate transcription factors in the MAPK signaling pathways downstream, while Tiron can suppress UVB-induced activation of these transcription factors.

Activated transcription factors c-Jun and c-Fos can be comprised of homodimer or heterodimer to bind to AP-1 binding sites in the promoter region of target genes to promote gene transcription [43, 44]. In addition, some reports indicated that phosphorylated ATF2 could be involved in AP-1-dependent transcription of the MMPs family as well [38]. So, we predicted the AP-1 binding sites in the MMP-1 and MMP-3 promoter region respectively. There is an AP-1 binding site (**-**74GCATGAGTCA**-**65) in the upstream of the transcription start site of MMP-1; at the same time, there is an AP-1 binding site (**+**127GGATGAGTCA**+**136) in the downstream of the transcription start site of MMP-3. Thus, luciferase reporter plasmids were constructed containing the AP-1 binding site sequences in MMP-1 and MMP-3 promoter region and then transfected into 293T cells. After treated with UVB, it can be observed a significant increase in their Luc activity. To further confirm effect of AP-1binding sites in the MMP-1 and MMP-3 promoter region on UVB-induced transcriptional activation, luciferase reporter plasmids containing mutated AP-1 binding site sequences in the MMP-1 and MMP-3 promoter region were constructed. After transfected into 293T cells, these mutated luciferase reporter plasmids indicated no difference in their Luc activity when treated with UVB. Therefore, the AP-1 binding sites in the MMP-1 and MMP-3 promoter region are indispensable for the expression of UVB-induced MMP-1 and MMP-3. On this basis, we also found that Tiron could inhibit UVB-induced Luc activity enhancement in a dose-dependent way.

Although it is still unclear that the directly targeting molecules of Tiron involve in the inhibition of MMP-1 and MMP-3 by UVB-induced in fibroblasts, the findings provide useful clues for further explore its target molecules in the process. Taken together, the development of novel MMPs inhibitors may be a promising strategy for skin photoageing. Our results demonstrate that Tiron is a potent inhibitor of the UVB-induced expression of MMPs via the inhibition of the MAPK/AP-1 signaling pathway in HDFs. Therefore, Tiron should be viewed as a potential therapeutic candidate for preventing and treating skin photoageing.

## Supporting Information

S1 Fig Expression of UVB-induced MMP-1 and MMP-3 in HDFs. A, HDFs were treated with UVB40 mJ/cm2. HDFs were harvested 0, 24, 48, 72h after the UVB treatment. The expression of MMP-1 and MMP-3 was assayed after the UVB treatment by western blotting. Protein expression levels of MMP-1andMMP-3 were normalized to that of GAPDH. B, The densitometric analysis for the data shown in (A) for MMP-1 and MMP-3 is shown. C-D, HDFs were exposed to UVB light with a total dose of 40 mJ/cm2. Cells were cultured 0, 24, 48, 72h after the UV exposure for total RNA extraction, and RT-PCR was performed later.
(TIF)

## Abbreviations

UV: ultraviolet
Tiron 4: 5-dihydroxy-1, 3-benzene disulfonic acid
ROS: reactive oxygen species
HDFs: human dermal fibroblasts
MMP: matrix metalloproteinase
AP: activator protein
SOD: superoxide dismutase
