# Supplementary figures and images for: Tiron Inhibits UVB-Induced AP-1 Binding Sites Transcriptional Activation on MMP-1 and MMP-3 Promoters by MAPK Signaling Pathway in Human Dermal Fibroblasts
Source: PLoS One. 2016 Aug 3;11(8):e0159998. doi: 10.1371/journal.pone.0159998 (PMC4972414; doi:10.1371/journal.pone.0159998)

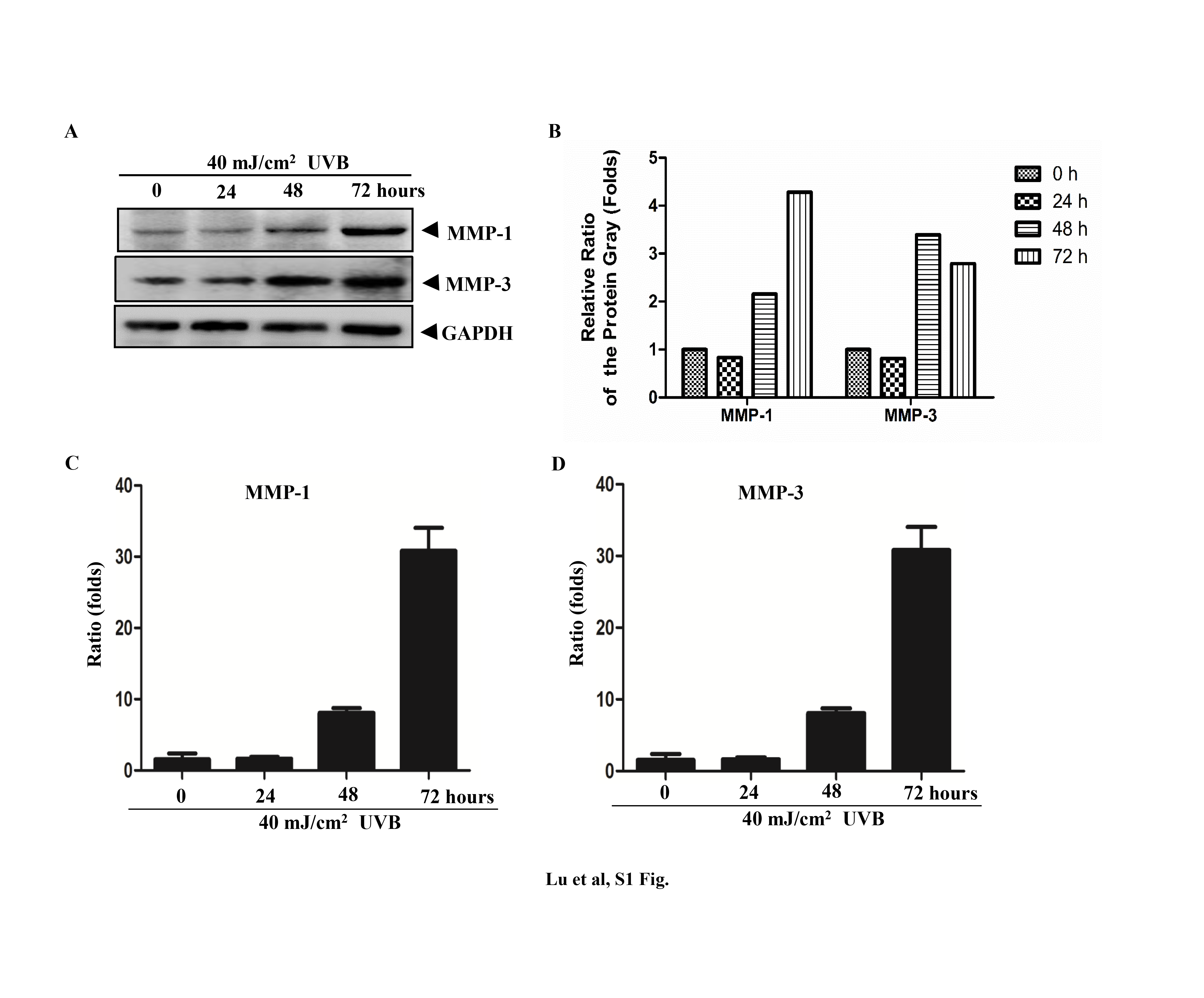

Supplement: S1 Fig — A, HDFs were treated with UVB40 mJ/cm2. HDFs were harvested 0, 24, 48, 72h after the UVB treatment. The expression of MMP-1 and MMP-3 was assayed after the UVB treatment by western blotting. Protein expression levels of MMP-1andMMP-3 were normalized to that of GAPDH. B, The densitometric analysis for the data shown in (A) for MMP-1 and MMP-3 is shown. C-D, HDFs were exposed to UVB light with a total dose of 40 mJ/cm2. Cells were cultured 0, 24, 48, 72h after the UV exposure for total RNA extraction, and RT-PCR was performed later. (TIF) [file pone.0159998.s001.tif]
